# Diagnostic testing of chronic wasting disease in white-tailed deer (*Odocoileus virginianus*) by RT-QuIC using multiple tissues

**DOI:** 10.1371/journal.pone.0274531

**Published:** 2022-11-16

**Authors:** Kate R. Burgener, Stuart S. Lichtenberg, Aaron Lomax, Daniel J. Storm, Daniel P. Walsh, Joel A. Pedersen

**Affiliations:** 1 Molecular and Environmental Toxicology Program, University of Wisconsin–Madison, Madison, Wisconsin, United Sates of America; 2 Department of Soil Science, University of Wisconsin–Madison, Madison, Wisconsin, United States of America; 3 Wisconsin Department of Natural Resources, Eau Claire, Wisconsin, United States of America; 4 U.S. Geological Survey, National Wildlife Health Center, Madison, Wisconsin, United States of America; National Institute of Allergy and Infectious Diseases, UNITED STATES

## Abstract

Chronic wasting disease (CWD) is a fatal prion disease affecting cervids (deer, elk, moose). Current methods to monitor individual disease state include highly invasive antemortem rectal biopsy or postmortem brain biopsy. Efficient, sensitive, and selective antemortem and postmortem testing of populations would increase knowledge of the dynamics of CWD epizootics as well as provide a means to track CWD progression into previously unaffected areas. Here, we analyzed the presence of CWD prions in skin samples from two easily accessed locations (ear and belly) from 30 deceased white-tailed deer (*Odocoileus viginianus*). The skin samples were enzymatically digested and analyzed by real-time quaking-induced conversion (RT-QuIC). The diagnostic sensitivity of the ear and belly skin samples were both 95%, and the diagnostic specificity of the ear and belly skin were both 100%. Additionally, the location of the skin biopsy on the ear does not affect specificity or sensitivity. These results demonstrate the efficacy of CWD diagnosis with skin biopsies using RT-QuIC. This method could be useful for large scale antemortem population testing.

## Introduction

Transmissible spongiform encephalopathies (TSEs) are infectious diseases caused by prions—protease-resistant proteins that convert endogenous host prion protein into misfolded conformers which easily aggregate into amyloid fibrils [1]. Prions resist inactivation by most means that are effective against conventional pathogens, and prion diseases are inevitably fatal once disease symptoms manifest [2–4]. Chronic wasting disease (CWD) is a TSE that affects cervids (deer, elk, moose) and is unique among prion diseases in that it affects populations that are largely undomesticated [5].

The current standard diagnostic methods for CWD testing in the United States rely on postmortem screening of obex or retropharyngeal lymph node (RPLN) biopsies by an enzyme-linked immunosorbent assay (ELISA) followed by confirmation via immunohistochemistry (IHC) [6]. Interpretation of IHC results requires a qualified pathologist and provides a qualitative measure of disease progression. Antemortem ELISA and IHC testing can be performed with rectoanal mucosa-associated lymph tissue (RAMALT) biopsies which can be as sensitive (true positive rate) as 68% and as specific (true negative rate) as 99% [7]. However, a RAMALT antemortem biopsy requires capture, local anesthesia, and surgical sampling of the rectum. The entire workflow is burdensome for wildlife agencies and stressful for subject animals.

An exciting potential alternative to these standard diagnostic approaches is the real-time quaking-induced conversion (RT-QuIC) assay. The RT-QuIC assay is an amyloid detection assay that exploits the capacity of prions to template the conversion of normally folded prion protein to misfolded conformers [8]. A suspect sample is incubated in a reaction mixture containing recombinant prion protein. The assay cycles between shaking and incubating the sample while continuously reading the fluorescence of thioflavin T (ThT) which binds to and is stabilized by amyloid fibrils [9], resulting in an increase in fluorescence intensity and a bathochromic shift in the emission spectrum maximum [10]. The RT-QuIC assay is a highly sensitive and specific *in vitro* assay for prions that can show results in a matter of hours [11]. Real-time fluorescence allows for a semi-quantitative analysis of amyloid formation. Time-to-threshold measurements and maximum detectable dilution are common outputs of the assay, as a rapid time to threshold or seeding activity at very high dilutions correspond to a high concentration of seeding material in the sample. The high sensitivity of the assay allows samples with low abundances of prions to be detected when conventional assays would not detect the presence of prions. Unlike the protein misfolding cyclic amplification (PMCA) assay, another *in vitro* conversion assay that uses brain homogenate as its cellular prion source, RT-QuIC does not produce inherently infectious material [12], does not require maintenance of a colony of (transgenic) animals for assay substrate, and requires far less bench time to produce interpretable results.

Wildlife and agricultural agencies have the need to monitor the infection status of both captive and wild herds in North America as the disease is spreading and not easily controlled [13–15]. Many states that have CWD-infected cervid populations, as well many states adjacent to infected populations, use hunter-harvested cervids for postmortem sampling to determine the extent and intensity of CWD on the landscape. Current hunter-harvested monitoring involves either field removal of RPLN or collection sites where cervid heads can be dropped off for RPLN or obex testing [16]. For hunter-harvested animal monitoring, collection sites often require the entire head, which is cumbersome in terms of both handling, storage, and disposal. A more readily accessible tissue for either ante- or postmortem diagnosis would increase ease and throughput of testing and reduce disposal costs. In cervids, CWD prions have been detected in non-neuronal/lymphatic tissues such as ear [17, 18], skin [19], skeletal muscle [20], blood [21], and eye [22], as well as in secretions and excreta such as saliva, urine, and feces [21, 23–25]. The skin is the most easily accessible organ, and prions have been detected in the skin of rodents [19, 26], humans [27], and cervids [18]. Highly reliable tissue for testing is necessary, which is why obex and RPLN are currently used for diagnostics. However, prion abundance in the same tissues can be uneven; for example, the left and right RPLNs on the same animal have been found to differ vastly in prion concentration [28].

In the present study, we aimed to determine if the relative concentrations of prion in multiple tissues from individual white-tailed deer (*Odocoileus viginianus*), as measured by RT-QuIC, are correlated with each other and examine their efficiency for potential use in ante- and post-mortem CWD detection. The individuals analyzed in this study were selected from a wild population, naturally infected with CWD. For this reason, exact time of infection is unknown and disease progression can only be ascertained from clinical signs and diagnostic assays. We chose this design to emulate conditions likely to be encountered in realistic diagnostic scenarios. The tissues studied include obex, RPLN, RAMALT, sclera, ear, and belly skin. Obex, RPLN, and RAMALT were chosen for analysis due to their standard use as postmortem and antemortem testing tissues. Sclera was chosen because of its proximity to the brain, where late-stage CWD amyloids are concentrated [22, 29]. Ear is comparatively easy to sample and ear clippings have been utilized for genotyping live deer. Detection of prions in skin suggests this tissue may represent a convenient target for antemortem testing. However, conventional methods of prion detection are not always sensitive enough to detect the low abundance of prions in peripheral tissues. To determine if skin can be used as a reliable substitute for diagnosing CWD-prion presence when using RT-QuIC, we optimized the extraction of ear and belly skin tissue for sensitivity and specificity. We developed a tissue preparation method to enzymatically degrade interfering biological molecules in the samples while also remaining cost effective for large scale testing. A subobjective of this study was to determine if a preferred sample location exists on the ear when using our preparation method. We analyzed the relative prion abundance at seven different locations on the ear pinna, including sites with varying amounts of cartilage.

## Materials and methods

### Tissue sample sources

White-tailed deer tissue samples were sourced from deceased collared animals that were a part of a CWD monitoring project conducted by the Wisconsin Department of Natural Resources (DNR). The deer had been captured, collared with a global positioning system tracking device, and anesthetized before an ear clipping and RAMALT biopsy were performed in accordance with the Wisconsin DNR institutional animal care policy (protocol 16-Storm-01) [30]. Samples were involved in a Wisconsin DNR study, and the permit granting agency for field site access is the DNR; therefore, no permits were required to complete this work. The ear clipping was used for *PRNP* genotyping, and RAMALT was used for confirmation of CWD status by IHC. All deer were released irrespective of CWD status and collected when their GPS collar indicated a lack of movement (death). After necropsy, either the obex or RPLN tissue were analyzed by IHC to assess CWD status at time of death. The other experimental tissues used in this study were harvested during necropsy. Some deer carcasses had been scavenged prior to tissue collection. Because of this, some tissues were unavailable for analysis for some animals.

Tissues sampled included brain, RPLN, RAMALT, sclera, ear, and belly skin. Thirty white-tailed deer (nine bucks, 21 does; all age classes) that died between 2019 and 2021 were used as the tissue sources for this study. At the time of collaring, 12 deer were IHC-positive for CWD (with one unsuccessful IHC reading), and at time of death 16 were CWD-positive by IHC.

Ten CWD-negative brain and tissue samples were sourced from a population of white-tailed deer culled for population control. The source population is geographically isolated from any known CWD cases, and the negative samples have previously been confirmed to be negative for prion seeding activity by both RT-QuIC and protein folding misfolding cyclic amplification.

### Whole ear sample sources

To determine if sample location on the ear affects RT-QuIC results, a second set of seven samples were taken from whole ears collected from eight white-tailed deer. These deer were sourced from the Wisconsin DNR as hunter-harvested specimens from the 2018 hunting season. Seven of the deer were CWD-positive and one was CWD-negative as assessed by IHC of RAMALT.

### Sample preparation

Obex samples were homogenized using bead beater (1 min, 4 m/s; Fisherbrand Bead Mill 24) with 0.7 mm diameter zirconia beads (BioSpec cat. no. 11079107zx) in 1× PBS (Fisher Scientific BP3991) to produce a 10% wt/v suspension. Samples were centrifuged (2 min, 3,000*g*), and the supernatants were collected. The supernatants were centrifuged again (3 min, 3,000*g*), aliquoted into 10 μL samples, and frozen at –80°C.

All other tissues were digested prior to analysis using the following protocol. A 10% wt/v solution of tissue was prepared in a solution of 1× PBS, 2 mM CaCl_2_ (Dot Scientific DSC20010-1000), and 0.25% wt/v collagenase A (Sigma-Aldrich 10103586001) [19, 22]. Samples were homogenized with a bead beater and zirconia beads as above. The samples were then shaken with a thermomixer (24 h, 45°C; Eppendorf ThermoMixer F1.5). After agitation and incubation, the samples were centrifuged (2 minutes, 3,000*g*), and the supernatants were retained. The supernatants were centrifuged again (3 min, 3,000*g*) to remove any small particulate matter, aliquoted, and frozen at –80°C.

### RT-QuIC

Samples were diluted in RT-QuIC sample buffer (0.1% SDS (Fisher Scientific BP166), 1% N-2 MAX Media Supplement (R&D Systems AR009) in 1× PBS). The reaction buffer consisted of 1× PBS, 170 mM NaI (LabChem LC246451), 1 mM EDTA (IBI Scientific IB70182), 0.1 mM Thioflavin T (Sigma SHBL4963), and 0.1 mg/mL recombinant hamster PrP (90–231), synthesized as previously described [12, 17]. For each replicate, 2 μL of sample in sample buffer was added to 98 μL of reaction buffer in individual wells of a black 96-well clear optical bottom plate (Thermo Fisher Scientific, 265301). The plates were sealed and incubated for 48 h at 50°C in a FLUOstar Omega plate reader (BMG) with 60 s double orbital shaking at 700 rpm followed by 60 s of rest. Fluorescent measurements were taken every 15 min with an 448–10 excitation filter and an 482–10 emission filter.

Obex and RPLN samples were analyzed at 10^−3^ to 10^−9^ dilutions in RT-QuIC sample buffer. The RAMALT samples were analyzed using 10^−3^ to 10^−7^ dilutions. Sclera, ear, and belly skin samples were analyzed from 10^−2^ to 10^−5^ dilution. All samples were analyzed using eight technical replicates and were considered positive if four or more replicates exceeded a threshold of 10 times the standard deviation of the baseline (the average of cycles 3–13) fluorescence before 40 h.

### Statistics

All statistics were performed with MATLAB and Statistics Toolbox release 2020b (The Math Works Inc., Natick, Massachusetts, United States). The Kruskal-Wallis *H*-test was used to test differences in median times to threshold for ear sample locations on whole white-tailed deer ear pinna. The Kruskal-Wallis *H*-test is a nonparametric variance analysis that tests by rank the null hypothesis that samples originate from the same population distribution. The Friedman test is a nonparametric repeated measures test that uses rank to test the null hypothesis that samples originate from the same population. It was used to test the ranges of time to threshold data for ear sample locations.

Spearman rank correlation is a nonparametric test that measures the correlation between two ranked variables. This statistical test, unlike the Pearson correlation, is not limited to linear relationships, but can be applied to any monotonic function. The Spearman rank correlation coefficient, rho (ρ), ranges from –1 to 1, where high positive values indicate a strong increasing monotonic relationship, low negative numbers indicate a strong decreasing monotonic relationship, and a value of 0 indicates lack of a relationship between the two variables.

Cohen’s kappa (κ) coefficient is a measure of agreement often used to compare two methods or assays. An advantage over a percent agreement calculation is that Cohen’s kappa considers the probability of agreement due to chance [31]. In this study Cohen’s kappa was used on a nominal dichotomous rating of positive or negative CWD status. The resulting κ can be a value up to 1 (total agreement), and confidence intervals are used to interpret agreement. Five semi-quantitative categories exist based on the value of κ: poor (κ < 0.2), fair (0.21 < κ > 0.4), moderate (0.41 < κ > 0.6), good (0.61 < κ > 0.8), and very good (0.81 < κ > 1.0) [32].

## Results & discussion

### Tissue samples

We acquired tissues postmortem from thirty white-tailed deer and analyzed them by RT-QuIC for the presence of prion seeding activity. The source of the deer is described in the Materials and methods section. Of the thirty individual deer in the study, 16 were judged positive and 14 were deemed negative by IHC analysis of obex tissue, RPLN tissue, or both. The organs and tissues studied were obex, RPLN, RAMALT, sclera, ear, and belly skin. We identify individual animals in this article by their prion protein gene (PRNP) 96-allele genotype, sex, and the order of necropsy. For example, individual G/G F 1 was the first deer to be necropsied, female, and homozygous for glycine at position 96. All available tissues were assayed by RT-QuIC in a dilution series with eight technical replicates for each dilution (S1 and S2 Tables). The sample was deemed positive for prion presence if at least four of the eight technical replicates indicated prion converting capacity (50%). Time to threshold was determined as the time from initiation of the reaction to that at which ThT fluorescence at 480 nm exceeded ten times the standard deviation of the fluorescence baseline (measurements from cycles three through thirteen). Not all organs and tissues were able to be harvested from some animals due to scavenging, decomposition, or the absence of excess tissue after mounting samples for IHC analysis. Negative control obex, RPLN, RAMALT, sclera, and ear tissue were tested alongside sample tissues (S3 Table). At the time of this work, no confirmed negative control belly skin tissue was available. However, all negative controls were remarkably consistent, and all tissues had an average amyloid formation incidence of less than 1/8. The time to thresholds of the amyloid formation in the control tissues were all much greater than their CWD-positive counterparts.

**Fig 1:**
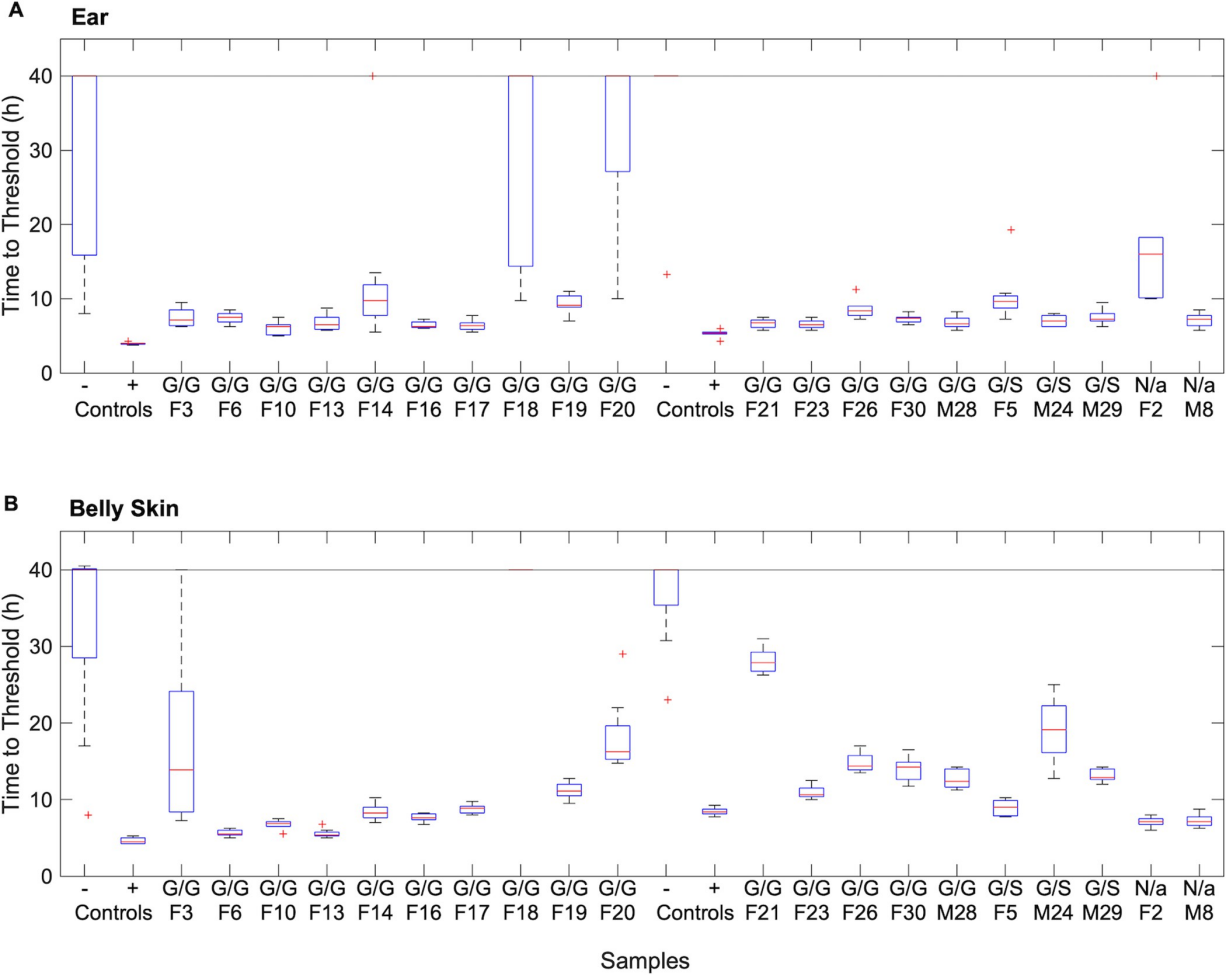
Real-time quaking-induced conversion (RT-QuIC) analysis of ear and belly skin tissue sampled from CWD-positive white-tailed deer. Box plots indicate the median with a horizontal red line, second and third quartiles with the box, and first and fourth quartiles with the whiskers. Statistical outliers are indicated by red crosses. Controls are known CWD-positive and negative obex run at 10^−3^ dilutions. The horizontal black line at 40 h indicates the end time of the assay. (A) Ear time to threshold results from samples at dilutions of 10^−2^. (B) Belly skin results from tissue samples at 10^−2^ dilutions.

Twenty-eight ears and thirty belly skin tags were analyzed by RT-QuIC for the presence of prion seeding activity (Fig 1). Relative to the disease status determined by IHC, one sample from ear tissue and one from belly skin from a single deer, ’G/G F 18’, produced an apparent false negative signal. The ear and belly skin samples produced no false positives (*n* = 8 and 10, respectively). Relative to disease status based on IHC analysis, the sensitivity of both ear and belly skin tested with RT-QuIC was 95%, while the specificity was 100%. The median time to threshold for each sample was used for statistical correlation analysis using a Spearman’s rank order correlation test (S4 and S5 Tables). Correlations between belly skin and RPLN (ρ = 0.653, *p* = 0.0045) and between belly skin and sclera (ρ = 0.756, *p* < 0.001) were strong. In contrast, strong correlations were not apparent between the median time to threshold for an individual’s ear and obex (ρ = –0.071), RPLN (ρ = 0.090), RAMALT (ρ = –0.072) or sclera (ρ = 0.426). Similarly, median time to threshold for belly skin samples did not strongly correlate with their obex (ρ = 0.544) or RAMALT (ρ = 0.1936) counterparts. Furthermore, obex samples did not strongly correlate with RPLN (ρ = 0.156), RAMALT (ρ = –0.367), or sclera (ρ = –0.400) tissues. Relative abundance of CWD-prion in RPLN and RAMALT was not strongly correlated (ρ = 0.258).

Ear and belly skin samples that were positive by RT-QuIC on average demonstrated seeding activity before 15 h; there were no false positives and only one false negative. In some samples, more than half the technical replicates indicated seeding activity at dilutions of 10^−5^ and 10^−6^ (S1 Table), indicating very high concentrations of prions in those samples. The slower time to threshold and absence of detection at higher dilutions for ear and belly skin tissues relative to lymphatic or neural tissues was expected. Another indicator of high prion abundance, as well as consistent methodology, is the number of technical replicates that showed prion converting ability in each sample. For the 20 deer determined to be CWD-positive by RT-QuIC, 95% of belly skin and 85% of ear samples were positive in all eight replicates at dilutions of 10^−2^. For diagnostic purposes, where only a positive/negative determination of disease status is sought, skin tissue appears to be a highly specific and sensitive sample.

Wide variations in the lowest dilution leading to amyloid formation among tissues from the same individual were common; ‘G/G F 16’ and ‘G/G F 19’, for example, both had RPLN seeding activity at dilutions higher than 10^−8^, but the corresponding lowest dilution of ear samples were 10^−6^ and 10^−2^, respectively (S1 Table). These results indicate no specific pattern of prion accumulation in the tested tissues of white-tailed deer in our sample.

### Ear sampling location

**Fig 2:**
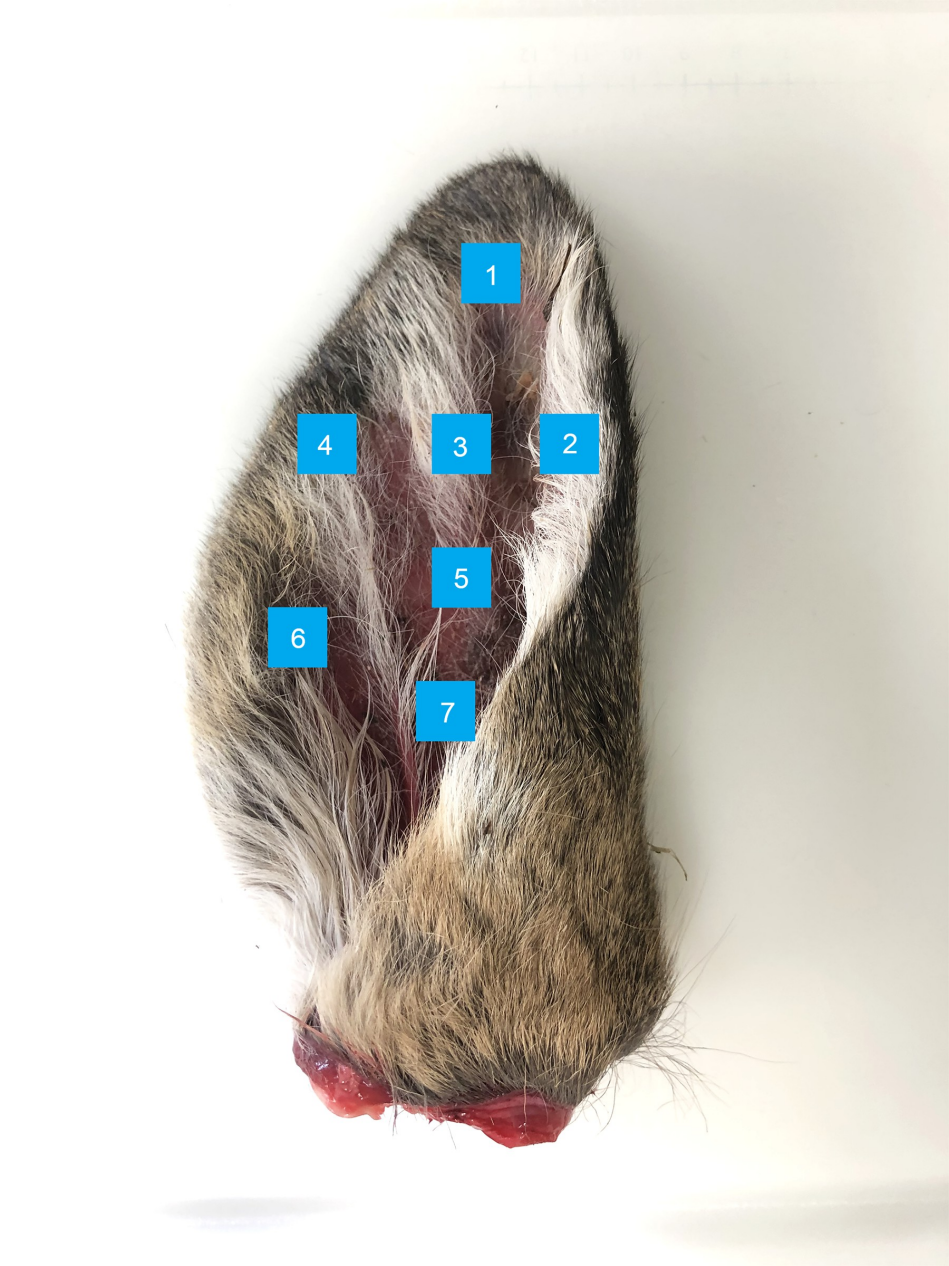
Ear sampling locations on white-tailed deer pinnae. Seven easily accessible locations in ear pinnae were tested for sensitivity and specificity on whole ears from seven CWD-positive deer. Deer tagging is usually performed on one or both ears and is primarily placed near location 7.

Ears from a separate group of eight white-tailed deer harvested in the 2018 Wisconsin white-tailed deer season were used to compare the relative abundance of prion converting activity in seven different ear pinna locations (Fig 2). Different locations were tested because, while the entire ear pinna of a white-tailed deer is vascularized, the amount of cartilage varies widely and could inhibit prion deposition in the tissue. Seven deer were RAMALT IHC-positive for CWD, and one deer was IHC-negative and used for a negative control. All CWD-positive ear samples demonstrated seeding activity at dilutions of 10^−2^ at each of the seven biopsy sites. Obex and ear samples of the negative control animal did not contain any detectable seeding activity. In addition to the hunter harvested negative control animal, ten negative control ears were analyzed and none demonstrated positive seeding activity (S1 Fig).

**Fig 3:**
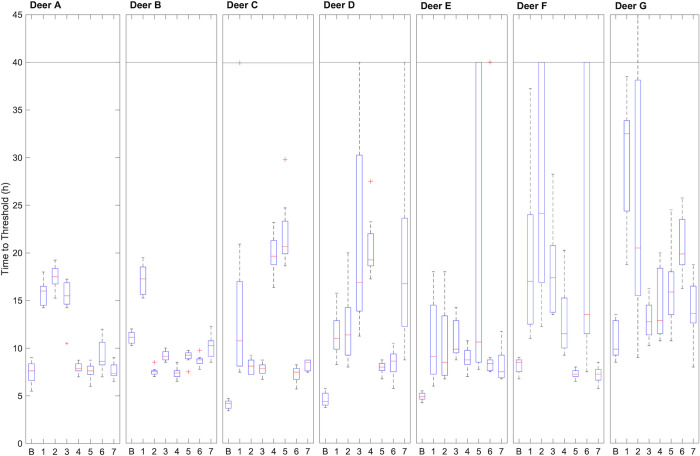
Time-to-threshold measurements of seven ear sample sites and obex of seven CWD-positive white-tailed deer. Box plots indicate the median with a horizontal red line, second and third quartiles with the box, and first and fourth quartiles with the whiskers. Statistical outliers are indicated by red crosses. The horizontal black line at 40 h indicates the end time of the assay. B is a 10^−3^ dilution of obex tissue from the indicated deer, and 1 through 7 indicate the sampling locations (see Fig 2) used to prepare 10^−2^ dilutions of ear skin tissue. The control deer is not shown.

The location of ear sampling on the pinnae of white-tailed deer does not seem to make a difference for detection of CWD prions in 10^−2^ dilutions of digested tissue homogenates (Fig 3). The time-to-threshold measurements and the lowest detectable dilution did not differ among sampling locations across ears (Kruskal-Wallis *H*-test, *p* > 0.05; S6 and S7 Tables). For individual ears, statistically significant differences existed in median time to threshold across sample sites (S6 Table). Overall, however, no one location stood out as better than the rest. As these deer were hunter-harvested, they may have presented no clinical signs before death, although the obex samples of these deer demonstrated prion seeding activity in dilutions from 10^−6^ to 10^−9^. Deer with prion seeding activity in obex at dilutions of 10^−9^ did not have statistically different ear seeding activity, indicating that for highly infected deer, specific patterns of prion accumulation do not exist in the ear among the locations tested.

A Spearman rank correlation analysis of the sample locations indicated a strong correlation existed between the time to threshold values of Location 1 and corresponding obex time to threshold values (ρ value of 0.857, *p* = 0.024). No strong correlations exist between the time to threshold for the obex and ear locations 2–6. This suggests that the relative abundance of prion in the tip of the ear (Location 1) may correlate with prion abundance in the obex. However, sample location 1 appeared consistently slower to reach threshold than the other sites (S6 Table) indicating that, for diagnostic purposes, other sampling sites may be more suitable. A Kruskal-Wallis *H* test indicated that no differences in time to thresholds existed among tested sample site locations on the ear (*p* = 0.368). In addition, the ranges of the time to threshold data are not statistically different for sample site locations when analyzed with a Friedman test (*p* = 0.496). These results indicate no favored location for sampling ear skin which means that sampling anywhere on the ear pinna produces sensitive and specific results. The flexibility in sample sites suggests that antemortem ear clip sampling need not be very precise and can be done by personnel without detailed knowledge of cervid ear anatomy. This idea also applies to postmortem testing by hunter-harvested samples; cutting a notch of ear skin from a carcass is far easier than removing the entire head for delivery to a drop off site.

A recent study investigated detection of CWD seeding activity in ear pinna from mule deer (*Odocoileus hemionus*) and white-tailed deer [18]. In that work, tissues were prepared by collagenase A enzymatic digestion followed by iron oxide magnetic extraction (IOME) bead enrichment and resulted in a sensitivity of 81% and specificity of 91% at the best sample location [18]. Our protocol uses only collagenase A enzyme digestion and resulted in a sensitivity of 95% and a specificity of 100%. Additionally, 88.6% of all 10^−2^ dilution ear samples demonstrated seeding activity in all eight technical replicates. These results apply to all testing locations, indicating that, by using our method of tissue preparation, high diagnostic sensitivity and specificity can be achieved with skin from anywhere on the ear pinna. In contrast, the recent study’s data suggested that 57.7% of initial results demonstrated seeding activity in all four of their technical replicates. The study retested any samples that were RPLN positive but did not have all four replicates indicate CWD presence in ear tissue; when tested again, 73.1% of their ear samples demonstrated seeding activity with RT-QuIC in all four technical replicates [18].

The same study concluded that the tip of the ear, equivalent of our sample Location 1 (Fig 2), was not an ideal location to biopsy as only one of ten ear samples reached threshold at dilutions of 10^−2^ or at 10^−1^ dilutions with IOME [18]. Our results suggest that for diagnostic purposes, the skin in the tip of the ear is adequate (all eight technical replicates showed consistent seeding activity, S7 Table). We note prion extraction using IOME beads allows prion detection in more dilute ear samples [18]; however, the cost of the beads needs to be factored in to determining the financial sustainability of their use in large-scale testing. In the same study, different sample locations on the ear were investigated, albeit at slightly different positions than in the present study. They concluded that the optimal sampling location was near our sample Location 3 (Fig 2), likely due to its proximity to the central auricular nerve [18]. Our results indicate no statistically significant difference in any results of the seven locations of an ear skin clip. We note that the previous study had a sample size of 56 composed of two different species of deer, mule deer (*n* = 50) and white-tailed deer (*n* = 6).

### Comparison of IHC and RT-QuIC results

The original 30 white-tailed deer from the tissues analysis were tested for CWD antemortem and postmortem by IHC. At the time of collaring, 12 deer were considered CWD-positive based on IHC analysis of RAMALT. At time of death, 16 deer were judged CWD-positive by IHC of obex, RPLN, or both. All tissues available from each deer were tested with RT-QuIC after enzymatic digestion (See Materials and methods). Deer were considered positive for prion converting activity if the majority of tissues exhibited seeding activity. The disease progression of CWD is such that robust seeding may be present in neural or lymphatic tissues but absent in peripheral tissues; by discriminating diagnostic status this way we ensure that animals with early-stage infections are correctly assessed as CWD-positive. Twenty deer were found to be positive for CWD by RT-QuIC (Table 1). Four notable discrepancies between the postmortem IHC and RT-QuIC results were apparent. Four animals deemed CWD negative by IHC exhibited strong prion seeding activity in all tested tissues, with their lowest detectable dilution and shown in Table 2.

**Table 1:** Assay results from white-tailed deer.

	IHC		RT-QuIC	Days between collaring and death	Scavenging
Sample ID	Antemortem	Postmortem	Postmortem		Determined by carcass retrieval team
G/G F 3	–	–	**+**	0	No
G/G F 6	**+**	**+**	**+**	21	No
G/G F 9	–	–	–	401	No
G/G F 10	–	–	**+**	747	No
G/G F 11	–	–	–	711	No
G/G F 13	**+**	**+**	**+**	28	Yes
G/G F 14	**+**	**+**	**+**	25	Yes
G/G F 16	**+**	**+**	**+**	102	No
G/G F 17	**+**	**+**	**+**	55	No
G/G F 18	Unsuccessful	**+**	**+**	53	Yes
G/G F 19	**+**	**+**	**+**	545	No
G/G F 20	**+**	**+**	**+**	533	No
G/G F 21	**+**	**+**	**+**	402	No
G/G F 23	**+**	**+**	**+**	536	No
G/G F 26	–	**+**	**+**	313	Yes
G/G F 30	–	**+**	**+**	1056	No
G/G M 1	–	–	–	192	Yes
G/G M 7	–	–	–	24	No
G/G M 15	–	–	–	375	No
G/G M 22	–	–	–	154	No
G/G M 28	**+**	**+**	**+**	159	Yes
G/S F 5	**+**	**+**	**+**	0	No
G/S F 12	–	–	–	1107	Yes
G/S F 27	–	–	–	1002	Yes
G/S M 24	**+**	**+**	**+**	216	No
G/S M 25	–	–	–	352	No
G/S M 29	–	–	**+**	994	No
N/A F 2	–	–	**+**	1034	Yes
N/A M 8	–	**+**	**+**	27	No
N/A F 4	–	–	–	0	No

Thirty white-tailed deer were RAMALT IHC tested after collaring, obex/RPLN IHC tested after death, and RT-QuIC tested using postmortem tissues (obex, RPLN, RAMALT, eye sclera, ear, and belly skin). Sample ID indicates genotype at the *PRNP* 96 allele, sex, and order of necropsy. One antemortem RAMALT test was unsuccessful. A sample was considered positive by RT-QuIC if the majority of tissues tested exhibited amyloid seeding (see S2 Table for additional replicate information).

**Table 2:** Disagreement between postmortem immunohistochemistry (IHC) and real-time quaking-induced conversion (RT-QuIC) results.

	IHC		RT-QuIC					
	Antemortem	Postmortem						
Sample ID	RAMALT	obex / RPLN	obex	RPLN	RAMALT	Sclera	Ear	Belly skin
G/G F 3	–	–		10^−6^ (8/8)	10^−6^ (4/8)	10^−4^ (8/8)	10^−4^ (4/8)	10^−2^ (8/8)
G/G F 10	–	–	10^−6^ (8/8)	10^−6^ (6/8)	10^−6^ (7/8)	10^−3^ (7/8)	10^−3^ (8/8)	10^−3^ (5/8)
G/S M 29	–	–	10^−7^ (6/8)	10^−7^ (4/8)	10^−7^ (4/8)	10^−5^ (8/8)	10^−2^ (8/8)	10^−4^ (8/8)
N/A F 2	–	–		10^−7^ (4/8)	10^−6^ (8/8)	10^−4^ (8/8)	10^−2^ (8/8)	10^−4^ (5/8)

Four IHC-negative deer tested positive using RT-QuIC in all tissues available. Blacked out spaces indicate that the tissue was not available to test. Dashes indicate that no seeding activity was detected at 40 h. Numbers indicate lowest dilution in which seeding activity was detected by RT-QuIC and the number of technical replicates is indicated inside the parentheses.

Initial postmortem CWD status by IHC indicated 53.3% (*n* = 16) of the deer were positive. However, after testing tissue samples with RT-QuIC, 66.7% (*n* = 20) were found to be CWD-positive. We note that in all four of the IHC false negatives, amyloid seeding activity was detected by RT-QuIC in obex, RPLN, and RAMALT in dilutions at least a million-fold smaller than the original samples. While the relative abundance of prions in skin tissue was lower than neural/lympthaic tissue, RT-QuIC analysis of skin allowed identification of CWD prions even in samples where IHC failed to identify CWD amyloids. Another advantage of RT-QuIC is that our protocol uses a tissue homogenate, which is more representative of the organ as opposed to a selective slice for IHC mounting. A homogenate reduces the likelihood of randomly testing a portion of a tissue lacking prions. A Cohen’s kappa coefficient was used to compare the IHC and RT-QuIC assay results. The resulting κ was 0.676 (classified as good) and had a 95% confidence interval of 0.417 to 0.935 which indicates that the disagreement between the IHC and RT-QuIC results are not completely due to chance.

#### Implications of findings

The tissue preparation protocol used in this study yields results with very high sensitivity and specificity for both ear and belly skin tissue samples. For ear clips, sampling location does not appear to make any diagnostic difference. Overall, the use of skin as a diagnostic measure of CWD status is far less invasive than current antemortem tests and is reliable with RT-QuIC. For diagnostic purposes, the IOME enrichment may be omitted without sacrificing sensitivity or specificity, as our method increases sensitivity from 81 to 95% and our specificity from 91 to 100% relative to a prior study employing IOME enrichment. Perhaps the most significant, although not altogether surprising, finding of this study was the poor sensitivity of IHC testing and the high number of false negatives that were found [33, 34]. This implies that CWD incidence and prevalence in white-tailed deer populations monitored using ELISA and IHC are underestimated. For hunter-harvested deer, diseased meat may be falsely considered free of CWD prions up to 13% of the time. A more sensitive prion assay such as RT-QuIC will provide a more accurate determination of the presence of CWD prions which may reduce exposure through reduced false negative results.

Our results suggest that antemortem testing may be accomplished by simply restraining and taking a skin biopsy, obviating the need for anesthesia or a trained technician to take a rectal biopsy. For postmortem testing, a skin sample would reduce the required drop-off criteria from an entire head to an ear, or minimally, less than a gram of tissue, which would dramatically decrease disposal and overall CWD testing costs, simplify sample preparation for sport hunters, and could increase voluntary participation in disease monitoring efforts. Disease monitoring of current CWD-infected cervid populations and populations adjacent to areas with CWD-infected herds is a high priority for state and federal wildlife agencies. A sensitive, high-throughput sample preparation method and detection assay will allow for significantly expanded monitoring efforts.

Future studies could determine the earliest time in disease progression at which prions can be detected by RT-QuIC in ear, skin, and other peripheral tissues. Early detection of prion diseases in animals facilitates timely implementation of proactive management decisions such as culling. Terminating sick animals may reduce transmission, ultimately slowing disease progression in the area [35]. An interesting consideration concerning the presence of prions in skin is that dead skin cells can comprise a significant portion of dust [36, 37]. Aerosol transmission of prions to cervids has been found to be more effective than oral exposure [38]. The presence of scrapie prions in dust has been reported [39]. The source of prions in the aerosolized particles was unknown; skin matter, feces, saliva, and urine were all suggested as possible sources [39]. For captive deer kept in close quarters, accumulation dead skin cells and their association with dust particulates may contribute to the rapid spread of CWD once captive facilities become infected. Oral grooming behavior occurs both in the wild and in captivity [40]. The presence of detectable prions in skin tissues suggests that skin may contribute to disease transmission, and the specific infectivity of prions in shed skin cells should be ascertained.

## Supporting information

S1 Fig Time-to-threshold measurements of ten negative control white-tailed deer ears at 10^−3^ dilutions. Box plots indicate the mean with a horizontal red line, second and third quartiles with the box, and first and fourth quartiles with the whiskers. Statistical outliers are indicated by red crosses. The horizontal black line at 48 h indicates the end time of the assay. Controls are known CWD-positive brain homogenates at 10^−3^ dilution and a control of just the reaction substrate. Two deer, C16 and D31, each had one out of eight technical replicates indicate seeding activity, though were not deemed CWD-positive because the number of replicates that turned on was less than four.
(TIF)

S1 Table Lowest dilution of seeding activity in tissue from 20 white-tailed deer determined to be CWD-positive by the real-time quaking-induced conversion (RT-QuIC) assay. Obex, retropharyngeal lymph node (RPLN), recto-anal mucosal-associated lymphatic tissue (RAMALT), sclera, ear, and belly skin tissues were analyzed in dilution series with RT-QuIC. Color saturation indicates the lowest dilution where at least four out of eight technical replicates demonstrated seeding activity.
(TIF)

S2 Table Number of replicates that indicated amyloid formation for all available tissues of 30 collared white-tailed deer. Obex, retropharyngeal lymph node (RPLN), recto-anal mucosal-associated lymphatic tissue (RAMALT), sclera, ear, and belly skin tissue were analyzed with RT-QuIC in technical replicates of eight.
(TIF)

S3 Table Number of amyloid formations in negative control runs. CWD-negative control obex, retropharyngeal lymph node (RPLN), recto-anal mucosal-associated lymphatic tissue (RAMALT), sclera, and ear tissue were tested numerous times. Each data represents the number of replicates (out of eight) in a RT-QuIC run that indicated amyloid formation and their respective time to thresholds (h) in parentheses. The average time to thresholds in all negative tissues exceed average time to thresholds of their CWD-positive counterparts. Ear skin had the highest number of replicates turn on, although the average number of amyloid formation incidents was 0.12 (below 1/8).
(TIF)

S4 Table Spearman rank correlation coefficients between tissues. Coefficients highlighted in blue indicate statistically significant (*p* < 0.01) correlations. Where RPLN is the retropharyngeal lymph node and RAMALT is the recto-anal mucosal-associated lymphatic tissue.
(TIF)

S5 Table Median time to threshold of 20 white-tailed deer ears and belly skin samples determined to be CWD-positive by the real-time quaking-induced conversion (RT-QuIC) assay. Time to threshold was determined by the time at which fluorescent signal reaches 10× the standard deviation of the threshold (defined by the average of fluorescent readings of cycles 3–13). The median values correspond with the real-time quaking-induced conversion (RT-QuIC) assay results from Fig 1(A) and 1(B).
(TIF)

S6 Table Median time to threshold of each ear sample location site and obex of the seven CWD-positive deer. Color saturations rank the ear sample locations of each individual deer, where the highest saturation (navy blue) indicates the fastest ear site location to exhibit seeding activity. Ranked saturation analyses indicate highest abundance in sites six and seven (Fig 2), although none of the locations differed significantly from the others (*p* > 0.05, Kruskal-Wallis *H*-test).
(TIF)

S7 Table Highest dilution of detectable seeding activity of each ear sample location site of the seven CWD-positive deer. Color saturations indicate the lowest detectable dilution where amyloid seeding activity was present in at least four of the eight technical replicates. None of the sites (Fig 2) were significantly different in lowest dilutions.
(TIF)
