# Supplementary figures and images for: Diagnostic testing of chronic wasting disease in white-tailed deer (Odocoileus virginianus) by RT-QuIC using multiple tissues
Source: PLoS One. 2022 Nov 16;17(11):e0274531. doi: 10.1371/journal.pone.0274531 (PMC9668146; doi:10.1371/journal.pone.0274531)

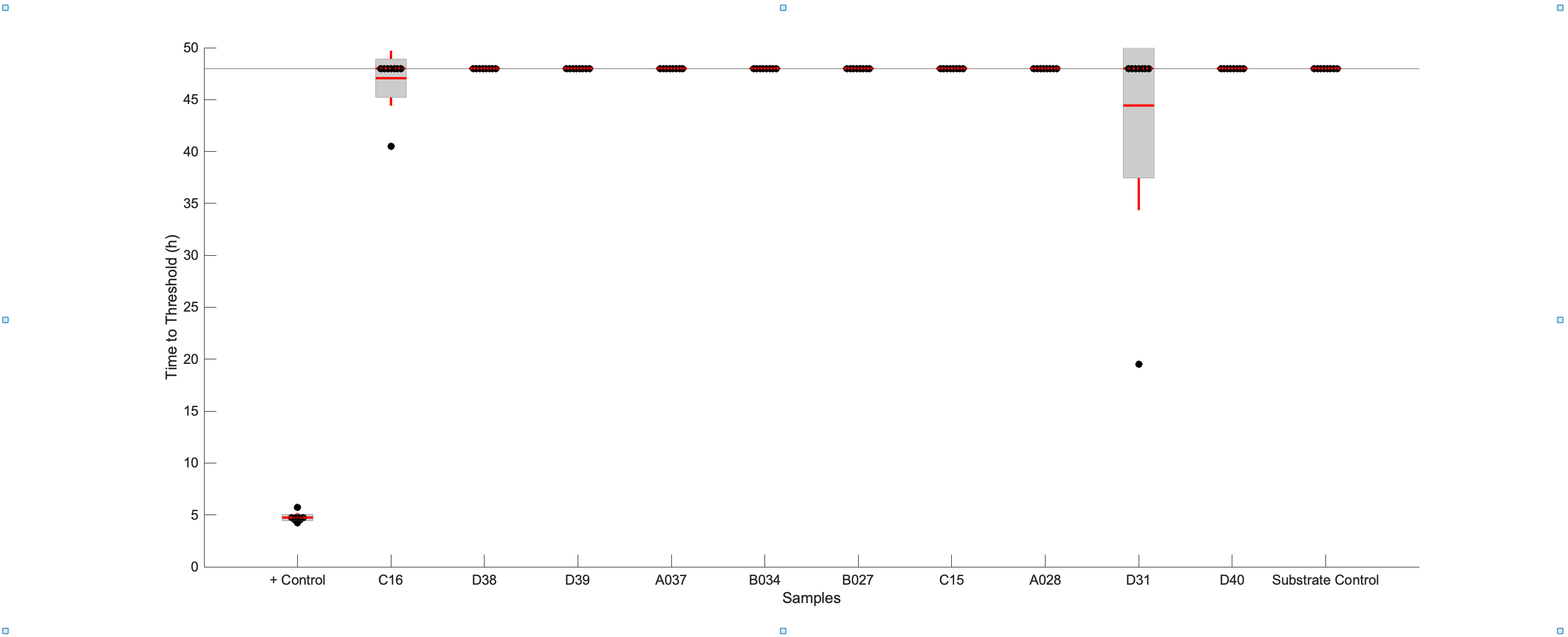

Supplement: S1 Fig — Box plots indicate the mean with a horizontal red line, second and third quartiles with the box, and first and fourth quartiles with the whiskers. Statistical outliers are indicated by red crosses. The horizontal black line at 48 h indicates the end time of the assay. Controls are known CWD-positive brain homogenates at 10−3 dilution and a control of just the reaction substrate. Two deer, C16 and D31, each had one out of eight technical replicates indicate seeding activity, though were not deemed CWD-positive because the number of replicates that turned on was less than four. (TIF) [file pone.0274531.s001.tif]

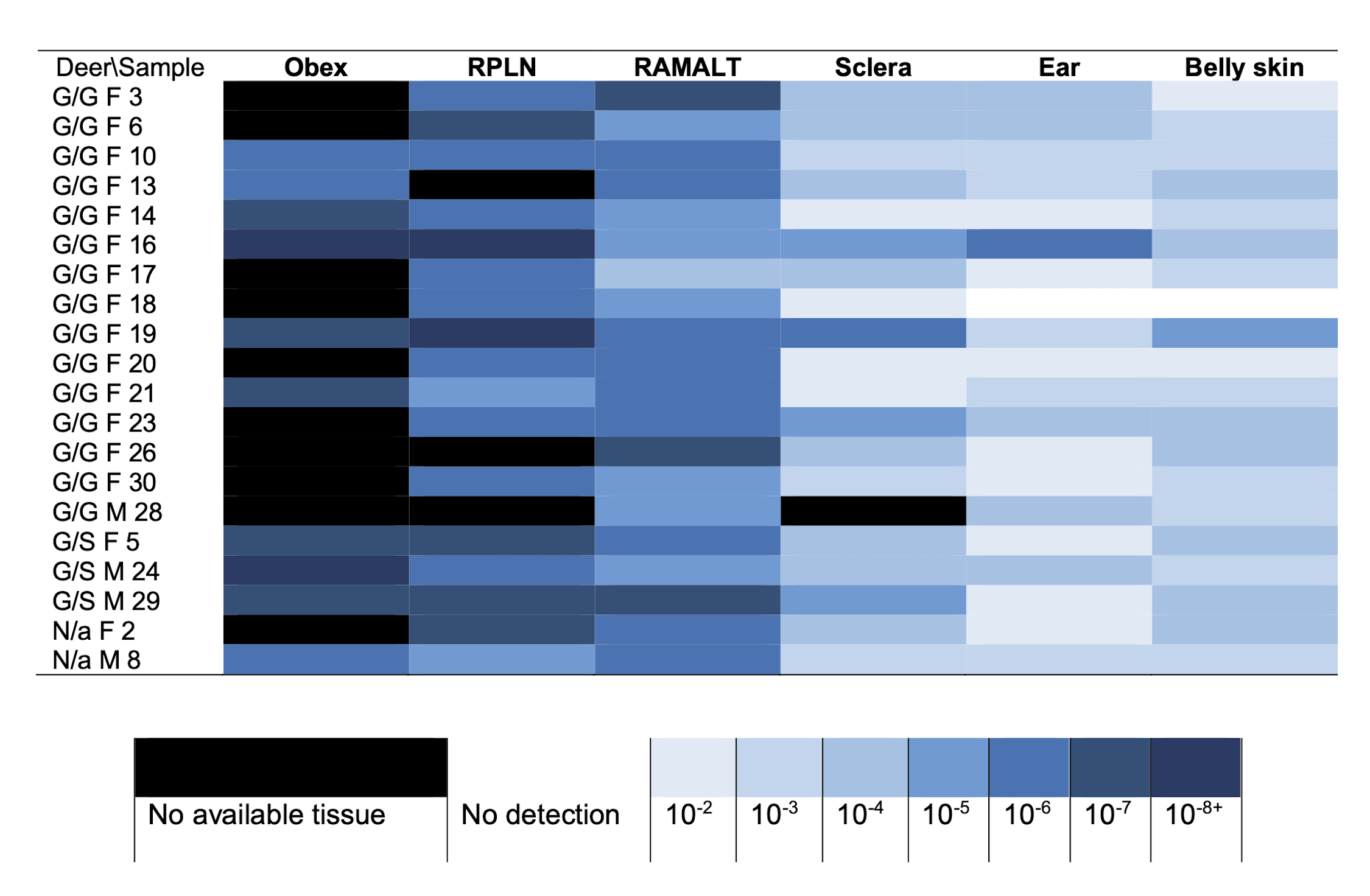

Supplement: S1 Table — Obex, retropharyngeal lymph node (RPLN), recto-anal mucosal-associated lymphatic tissue (RAMALT), sclera, ear, and belly skin tissues were analyzed in dilution series with RT-QuIC. Color saturation indicates the lowest dilution where at least four out of eight technical replicates demonstrated seeding activity. (TIF) [file pone.0274531.s002.tif]

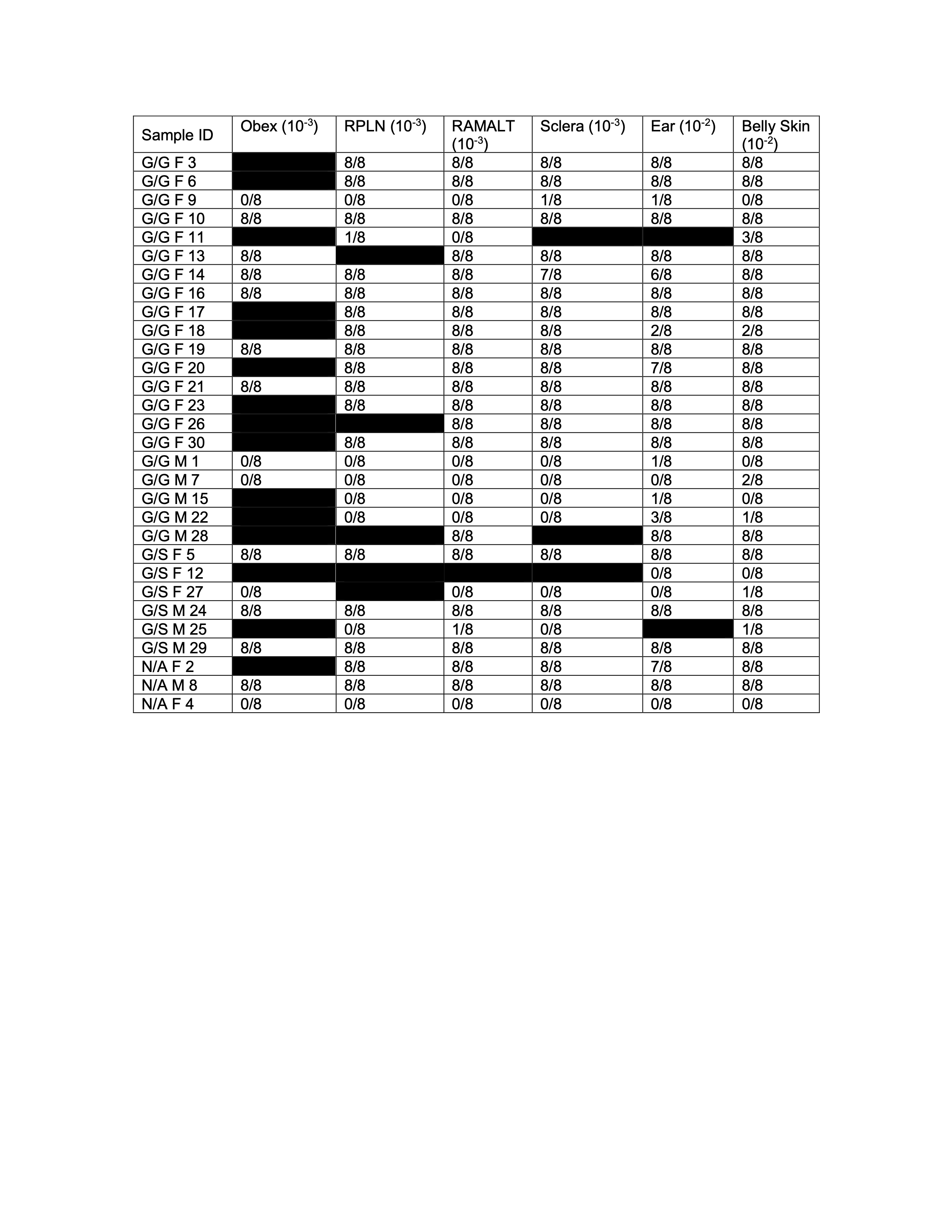

Supplement: S2 Table — Obex, retropharyngeal lymph node (RPLN), recto-anal mucosal-associated lymphatic tissue (RAMALT), sclera, ear, and belly skin tissue were analyzed with RT-QuIC in technical replicates of eight. (TIF) [file pone.0274531.s003.tif]

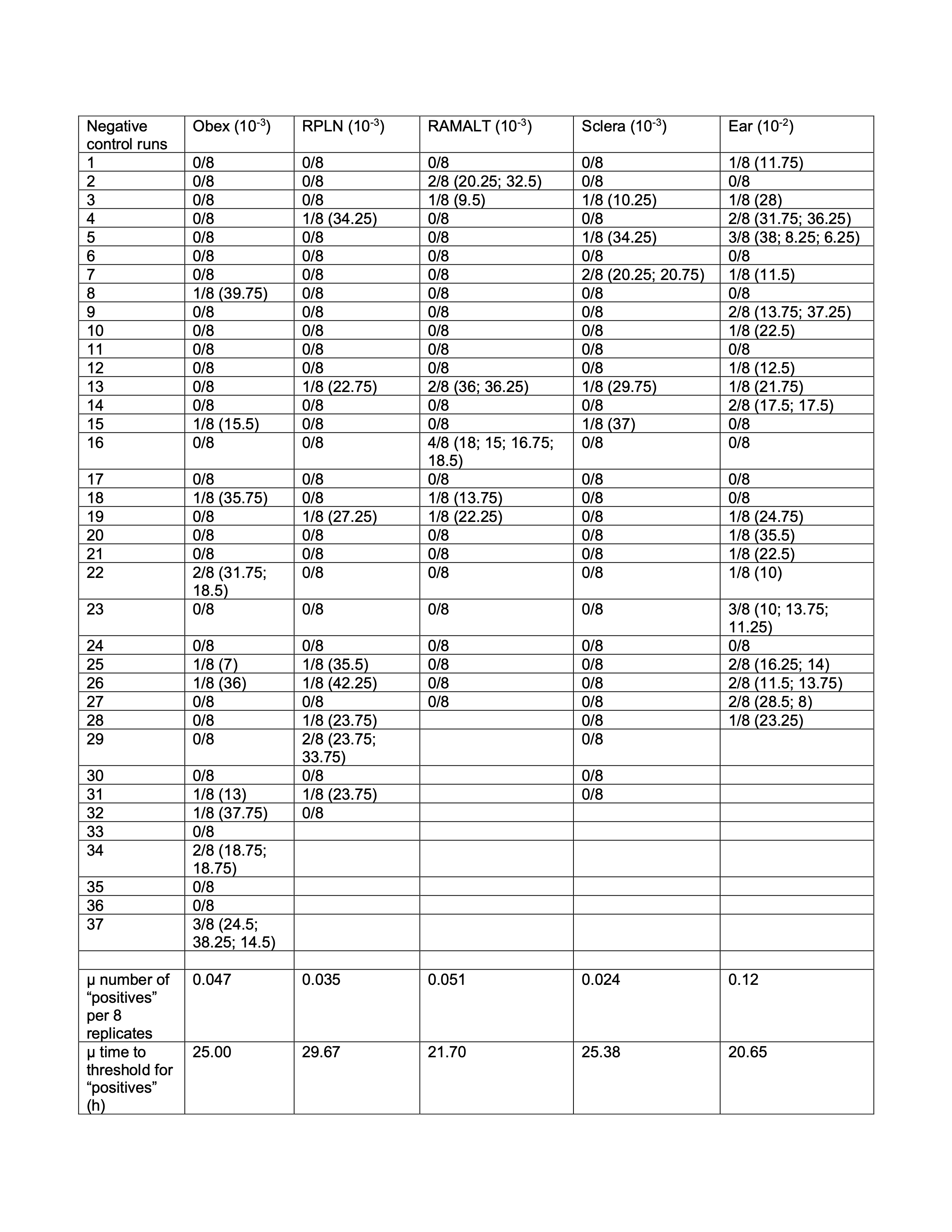

Supplement: S3 Table — CWD-negative control obex, retropharyngeal lymph node (RPLN), recto-anal mucosal-associated lymphatic tissue (RAMALT), sclera, and ear tissue were tested numerous times. Each data represents the number of replicates (out of eight) in a RT-QuIC run that indicated amyloid formation and their respective time to thresholds (h) in parentheses. The average time to thresholds in all negative tissues exceed average time to thresholds of their CWD-positive counterparts. Ear skin had the highest number of replicates turn on, although the average number of amyloid formation incidents was 0.12 (below 1/8). (TIF) [file pone.0274531.s004.tif]

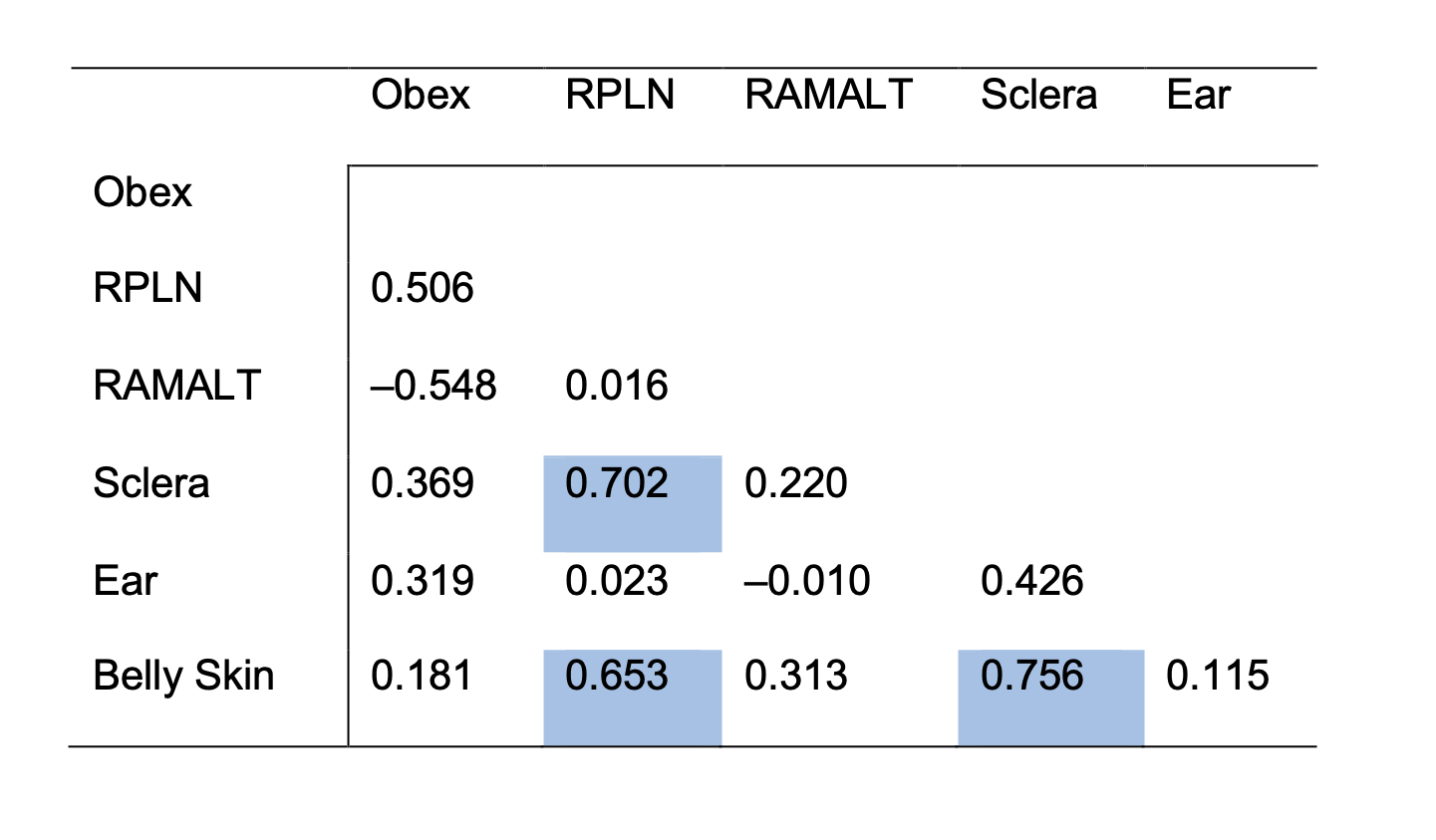

Supplement: S4 Table — Coefficients highlighted in blue indicate statistically significant (p < 0.01) correlations. Where RPLN is the retropharyngeal lymph node and RAMALT is the recto-anal mucosal-associated lymphatic tissue. (TIF) [file pone.0274531.s005.tif]

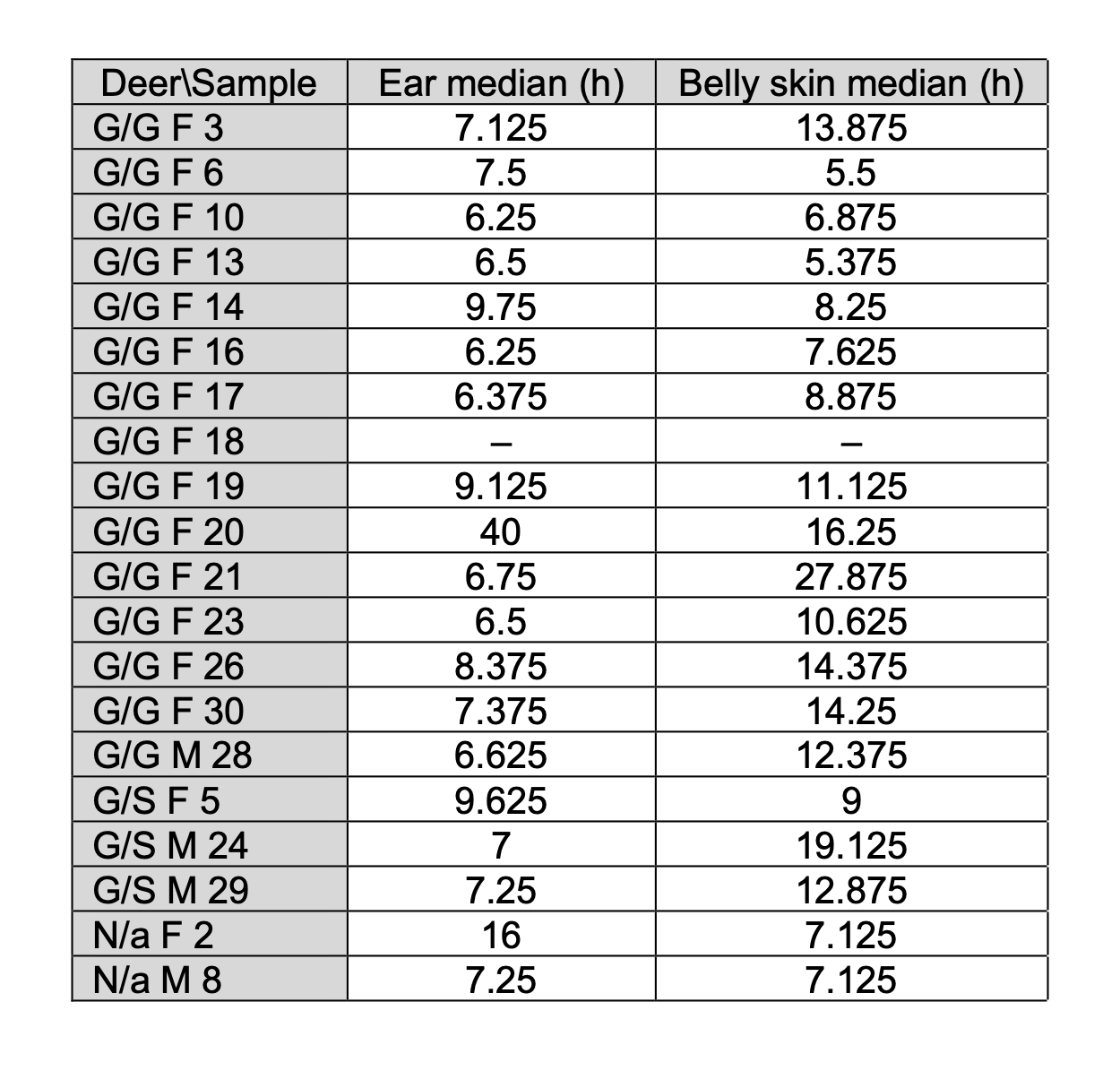

Supplement: S5 Table — Time to threshold was determined by the time at which fluorescent signal reaches 10× the standard deviation of the threshold (defined by the average of fluorescent readings of cycles 3–13). The median values correspond with the real-time quaking-induced conversion (RT-QuIC) assay results from Fig 1(A) and 1(B). (TIF) [file pone.0274531.s006.tif]

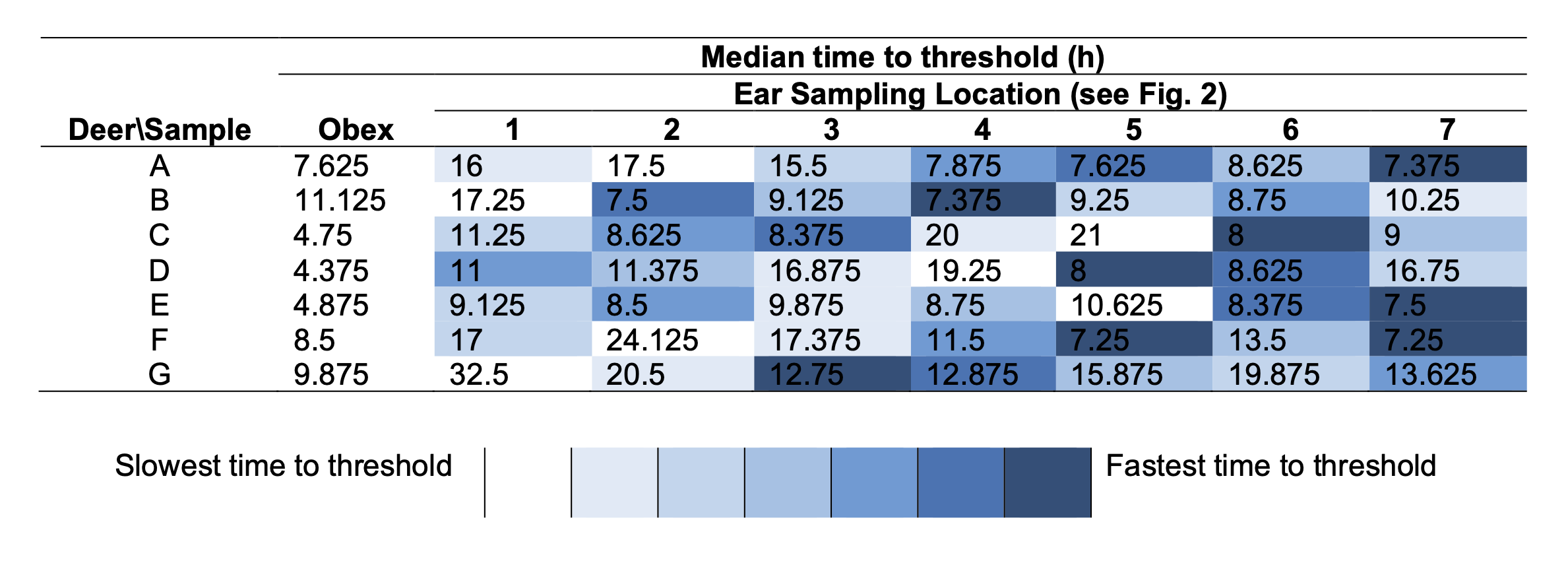

Supplement: S6 Table — Color saturations rank the ear sample locations of each individual deer, where the highest saturation (navy blue) indicates the fastest ear site location to exhibit seeding activity. Ranked saturation analyses indicate highest abundance in sites six and seven (Fig 2), although none of the locations differed significantly from the others (p > 0.05, Kruskal-Wallis H-test). (TIF) [file pone.0274531.s007.tif]

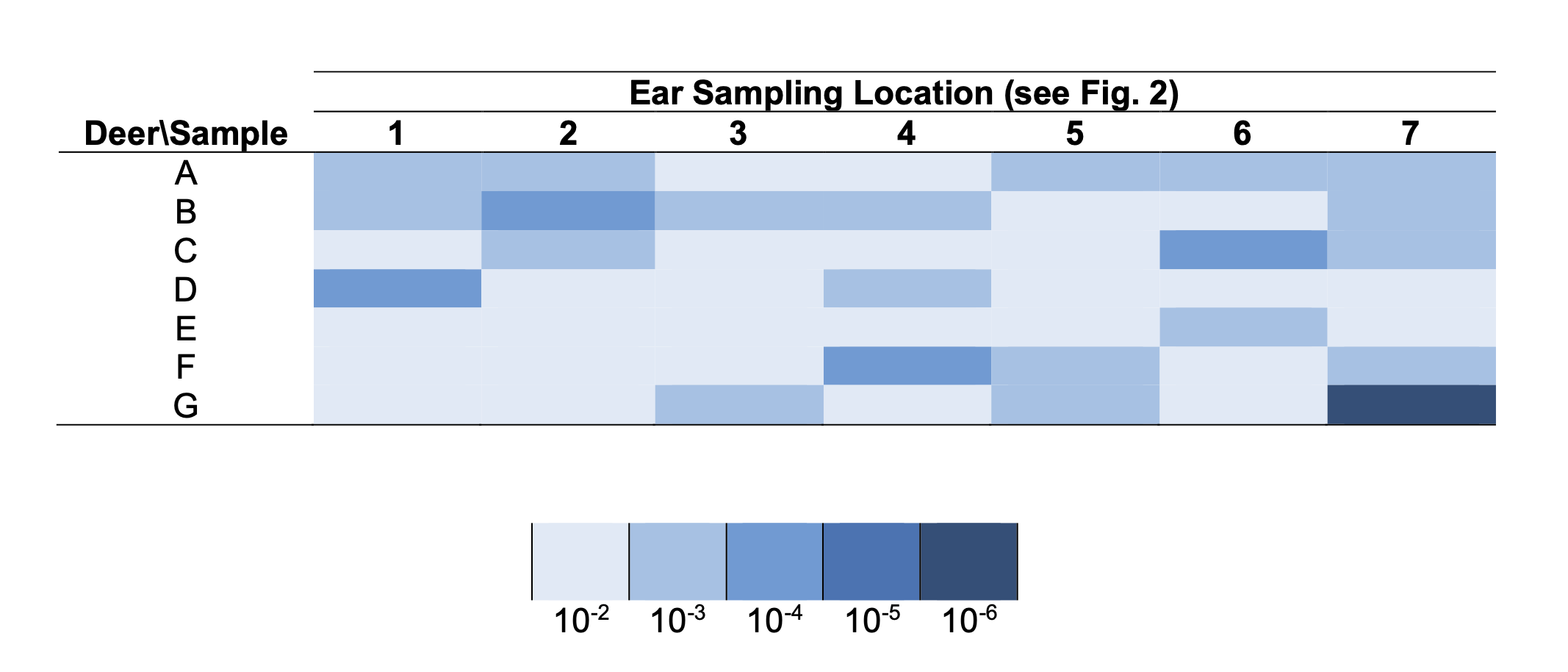

Supplement: S7 Table — Color saturations indicate the lowest detectable dilution where amyloid seeding activity was present in at least four of the eight technical replicates. None of the sites (Fig 2) were significantly different in lowest dilutions. (TIF) [file pone.0274531.s008.tif]
